# Gorlin–Goltz Syndrome: A Case Report and Literature Review with *PTCH1* Gene Sequencing

**DOI:** 10.1055/a-2096-3536

**Published:** 2023-08-02

**Authors:** Hyo Seong Kim, Seung Heo, Kyung Sik Kim, Joon Choi, Jeong Yeol Yang

**Affiliations:** 1 Department of Plastic and Reconstructive Surgery, Myong-ji Hospital, Deokyang-gu, Goyang, Republic of Korea; 2 Division of Pediatric Plastic Surgery, Seoul National University Children's Hospital, Jongno-gu, Seoul, Republic of Korea

**Keywords:** Gorlin–Goltz syndrome, *PTCH1* gene, basal cell carcinoma syndrome

## Abstract

Gorlin–Goltz syndrome, also known as nevoid basal cell carcinoma syndrome, is an autosomal dominant disease characterized by multisystemic developmental defects caused by *pathogenic variants such as patched-1* ( *PTCH1* ) gene variants and/or SUFU gene variants. The presence of either two main criteria or one major and two minor criteria are required for the diagnosis of Gorlin–Goltz syndrome. Recently, a major criterion for molecular confirmation has also been proposed. In this article, we report the case of an 80-year-old male who was admitted at our department for multiple brown-to-black papules and plaques on the entire body. He was diagnosed with Gorlin–Goltz syndrome with clinical, radiologic, and pathologic findings. While the diagnosis was made based on the clinical findings in general, confirmation of the genetic variants makes an ideal diagnosis and suggests a new treatment method for target therapy. We requested a genetic test of PTCH1 to ideally identify the molecular confirmation in the hedgehog signaling pathway. However, no pathogenic variants were found in the coding region of PTCH1, and no molecular confirmation was achieved.

## Introduction

Gorlin–Goltz syndrome, also known as nevoid basal cell carcinoma syndrome (NBCCS), is an autosomal dominant illness characterized by multisystemic developmental defects caused by *pathogenic variants such as patched-1* ( *PTCH1* ) gene and/or SUFU gene variants. It is predicted to affect 1 in 30,827 to 1 in 256,000 people globally, with a preference for Caucasians. However, it affects both men and women equally. 1 2 Jarish and White originally described the syndrome in 1894, and it was later dubbed Gorlin–Goltz syndrome when Gorlin and Goltz gathered its signs and symptoms. 3 4

The presence of either two main criteria or one major and two minor criteria is required for the diagnosis of Gorlin and Goltz syndromes ( Table 1 ). Recently, a major criterion of molecular confirmation has been proposed. 1 3 5 6

**Table 1:** Diagnostic criteria for nevoid basal cell carcinoma syndrome (adapted from Bresler et al 1 )

Major criteria
1. Basal cell carcinoma before 20 years of age or excessive numbers of basal cell carcinomas out of proportion to prior sun exposure and skin type
2. Keratocystic odontogenic tumor before 20 years of age
3. Palmar or plantar pitting
4. Lamellar calcification of the falx cerebri
5. Medulloblastoma, typically desmoplastic
**Minor criteria**
1. Rib abnormalities
2. Other specific skeletal malformations and radiologic changes (i.e., vertebral anomalies,
kyphoscoliosis, short fourth metacarpals, postaxial polydactyly)
3. Macrocephaly
4. Cleft lip or palate
5. Ovarian or cardiac fibroma
6. Lymphomesenteric cysts
7. Ocular abnormalities (i.e., strabismus, hypertelorism, congenital cataracts, glaucoma, coloboma)

In the view of molecule alterations, NBCCS is thought to be caused by variants in the components of the highly conserved hedgehog signaling system, which results in constitutive signaling activity, with the majority of variants occurring in the patched ( *PTCH1* ) gene on chromosome 9q22.3. 1 7 PTCH1, a membrane-bound protein, maintains smoothened (SMO) in an inactive/unphosphorylated form during inactive signaling, leaving it vulnerable to endocytosis and degradation. Therefore, the GLI proteins, which are transcription factors required for the activation (or repression) of pathway-dependent genes, cannot be activated. 1 8 9 Additionally, the protein suppressor of fused (SUFU, encoded by the SUFU gene), which is a component of the corepressor complex, contributes to the negative regulation of GLI factors by providing extra negative regulation through direct binding. 1 10 The binding of hedgehog ligands (including Indian hedgehog, desert hedgehog, and the most common sonic hedgehog [SHH]) to PTCH1, the hyperphosphorylation of SMO causes GLI to return to the nucleus. 1 9 This causes transcriptional modifications and a variety of downstream consequences during nearly every stage of the development. Under pathogenic circumstances, constitutive stimulation of the hedgehog signaling pathway as a result of variants in essential regulatory proteins results in tumor cell proliferation. 1 11 According to recent articles, a custom HaloPlex panel encompassing genes implicated in hedgehog-related pathways, such as PTCH1, PTCH2, SHH, SUFU, SMO, GLI1, GLI2, and GLI3, has been developed. 5 12

In this pathway, loss of function variants in PTCH1 are thought to occur in up to 70% of patients who fulfill diagnostic criteria for Gorlin–Goltz syndrome. Interestingly, the patients with SUFU variant appear to have a lower incidence of major criteria including keratocystic odontogenic tumor and have a greater risk of desmoplastic medulloblastoma that is more fatal. 1 PTCH2 variants have also been suggested to be a molecular cause of the syndrome, but they are still a topic of discussion because not enough data supporting hypothesis.

Therefore, a multicenter approach is necessary for the diagnosis and treatment of patients with Gorlin–Goltz syndrome, since a variety of clinical attitudes may be noticed during the course of a lifetime. Ideally, genetic consultation is critical and a gene variants test can provide the definitive diagnosis.

In this article, we report a case of an 80-year-old man who was admitted at our department for multiple brown-to-black papules and plaques on the entire body, including the scalp, face, chest, back, and bilateral lower extremities, and was diagnosed with Gorlin–Goltz syndrome based on the clinical, radiologic, and pathologic findings. Furthermore, we requested a genetic test to ideally identify the molecular confirmation in the hedgehog signaling pathway.

## Case

**Fig. 1:**
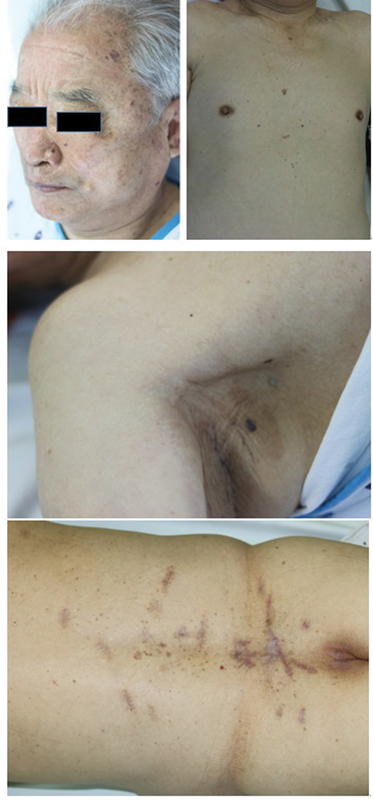
Multiple brown-to-black papules and plaques, suggestive of basal cell carcinoma on face, right axilla, chest, back, left lower leg.

An 80-year-old male presented to our plastic and reconstruction department with numerous brown-to-black papules and plaques over the entire body, including the scalp, face, chest, back, and bilateral lower extremities. These were presumed to be basal cell carcinomas, which had been present before the age of 20, but became more numerous and enlarged before the first visit ( Fig. 1 ).

In his history, he was known to have undergone surgery for a keratocystic odontogenic tumor at the right angle of the mandible 30 years ago.

**Fig. 2:**
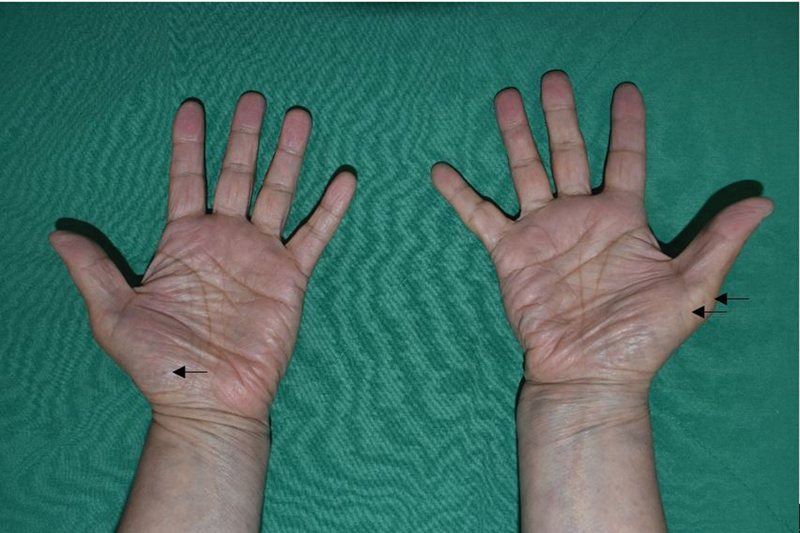
Bilateral hands. Multiple palmar pits (arrows).

On physical examination, three or more punctiform, brownish-black depressions on both hands, suggestive of palmar pitting and multiple, elastic, and hard skin cysts, were observed in both hands ( Fig. 2 ). An increased intercanthal distance (40 mm) was noted on facial examination, suggestive of orbital hypertelorism.

**Fig. 3:**
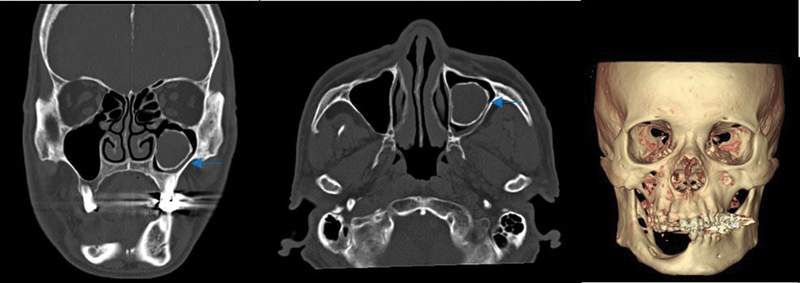
Radiopaque tumor showing a large well-defined unilocular lesion with corticated margins of approximately 3.1 cm × 3.0 cm involving the left maxillary sinus (arrow).

**Fig. 4:**
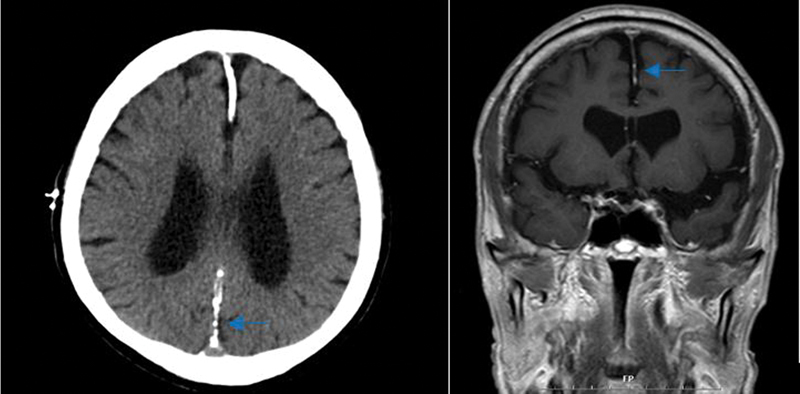
Dense linear calcifications along the falx cerebri (arrow). Mottled or intermittent dural calcification along both tentorial lining and bilateral high cerebral convexity.

Additional imaging tests were advised for detailed radiological assessment. Traces of surgical removal of keratocystic odontogenic tumors were found on the right angle of the mandible, and a radiopaque tumor showing a large, well-defined unilocular lesion with corticated margins of approximately 3.1 cm × 3.0 cm involving the left maxillary sinus was discovered on computed tomography ( Fig. 3 ). Calcification of the falx cerebri was incidentally found on brain magnetic resonance imaging, but did not show any cognitive impairment or other brain dysfunction ( Fig. 4 ). The ribs, limbs, and cervical spine radiographs showed no skeletal abnormalities, and there was no evidence of metastasis on the positron emission tomography scan.

The patient underwent 11 surgeries for tumor resection under general or local anesthesia because the tumors were numerous to be removed at once and continued to recur. All of the masses were totally excised with primary safety margin of 5mm, and under intraoperative frozen section biopsy, additional resection was performed until the tumor-free margin was confirmed. The specimens were confirmed to be basal cell carcinoma, invading the deep dermis at permanent pathology. The specimens were confirmed to be basal cell carcinoma, invading the deep dermis.

**Fig. 5:**
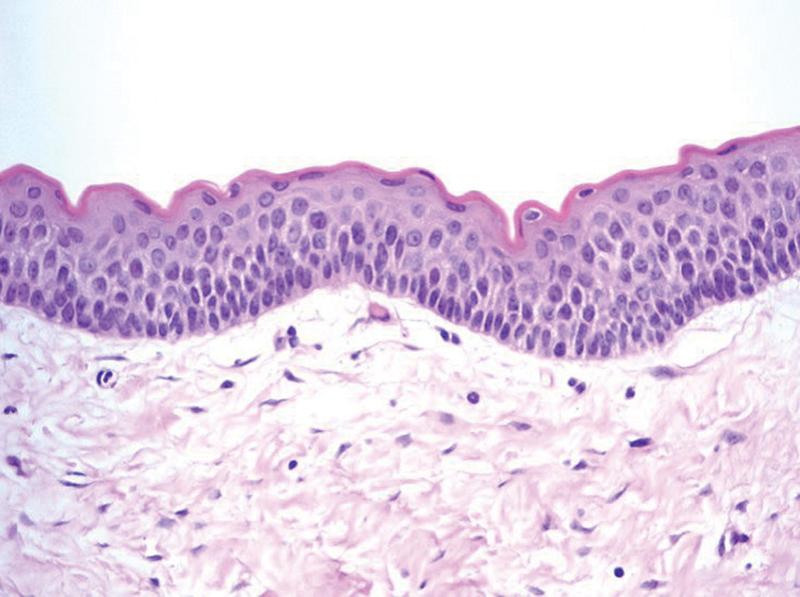
The cystic mass is lined by flat-appearing stratified squamous epithelium, showing the corrugated parakeratotic luminal surface with cuboidal to columnar palisading basal cells, consistent with odontogenic keratocyst (hematoxylin and eosin stain, × 400).

The radiopaque tumor in the left maxilla was removed using the Caldwell-Luc operation. On histopathological examination, the specimen was lined by flat-appearing stratified squamous epithelium, showing a corrugated parakeratotic luminal surface with cuboidal to columnar palisading basal cells, consistent with odontogenic keratocysts ( Fig. 5 ).

Based on the above clinical, radiologic, and histopathologic findings, Gorlin–Goltz eponymous syndrome was diagnosed. However, a genetic test was requested to ideally find the molecular confirmation in the hedgehog signaling pathway.

To confirm the presence of a genetic variant, blood sample was collected from the Department of Laboratory Medicine at Myongji Hospital, and the PTCH1 variant test was requested to the affiliated Seoul Clinical Laboratories (SCL), and DNA sequencing was performed through Sanger sequencing with the patient's signed consent. In this method, The DNA sample to be sequenced is combined in a tube with primer, DNA polymerase, and DNA nucleotides (dATP, dTTP, dGTP, and dCTP). A computer reads each band of the capillary gel, in order, using fluorescence to call the identity of each terminal ddNTP. In short, a laser excites the fluorescent tags in each band, and a computer detects the resulting light emitted. In conclusion, we were informed from Seoul Clinical Laboratories (SCL) that no variants were detected in the coding region of *PTCH1* gene while two polymorphisms were found.

## Discussion

The diagnosis of NBCCS requires the presence of two major criteria or one major and two minor criteria ( Table 1 ), or one major criterion with confirmation of a genetic variant in the molecular pathway. This patient was clinically diagnosed with Gorlin–Goltz syndrome with multiple early onset BCCs, palmar pits, calcification of the falx cerebri, and keratocystic odontogenic tumor. Lastly, the presence of orbital hypertelorism and multiple cysts on both hands that were pathologically undiagnosed met the minor criteria.

Odontogenic keratocysts occur in up to 75% of patients with Gorlin–Goltz syndrome. They are frequently discovered inadvertently during radiological tests and may be the first manifestation of the disease. In addition, tumor recurrence rates reaching up to 60% have been reported. 3 13 In this patient, the odontogenic keratocyst in the left maxillary sinus occurred even after the surgical removal of the odontogenic keratocyst in the right mandibular angle, confirming that the incidence and the high recurrence rates. Therefore, continuous radiologic follow-up is required.

The Bazex–Dupre–Christol syndrome, Muir–Torre syndrome, Rombo syndrome, multiple papular trichoepitheliomata, and xeroderma pigmentosum should all be considered as the differential diagnoses. In addition to multiple BCCs, Bazex–Dupre–Christol syndrome is marked by follicular atrophoderma, hypotrichosis, and hypohidrosis. Muir-Torre syndrome is a genodermatosis characterized by the presence of multiple sebaceous adenomas, multiple keratoadenomas, and gastrointestinal cancers, which are not seen in Gorlin–Goltz syndrome. Rombo syndrome is characterized by vermiculate atrophoderma, hypotrichosis, cyanotic erythema of the hands and feet, numerous BCCs, and trichoepitheliomata. 3 14

Although Gorlin–Goltz syndrome has provided critical genetic clues in the hedgehog signaling pathway, 80% of sporadic keratocystic odontogenic tumor reports are due to variants in PTCH1. 5 15 In this case, no variants were detected in the coding region of the *PTCH1* gene, while it cannot be completely excluded that PTCH1 variants are often undetected in some patients because routine Sanger sequence analysis is labor-intensive and time-consuming. 5 16 Also this analysis has limiting factors for the negative genetic test, such as no gene-targeted deletion/duplication analysis, no sequencing of the intronic regions and the possibility of mosaicism detected in blood. In addition, although the possibility of variants in other genes cannot be rules out, additional genetic testing other than PTCH1 through the SCL was a limiting factor.

In the treatment method, conservative early tumor resection is necessary. However, patients with NBCCS may require a significant number of excisions, owing to the large tumor burden that is frequently observed. Because of the vast number of excisions required, individuals may suffer significant deformities. Therefore, topical application of various medicines, such as 5-fluorouracil and imiquimod, are examples of conventional therapies for localized diseases that are not surgical in nature. 1 Recently, vismodegib, a small-molecule drug that binds to and directly inhibits SMO, was licensed by the Food and Drug Administration for the treatment of basal cell carcinoma that has returned, progressed locally, or spread across the body. 17 However, the possibility of complications of vismodegib should be carefully considered and the general condition and situations of the patients should be understood. Above all else, frequent multidisciplinary surveillance is necessary for individuals with established NBCCS and sun protection is an important preventative care measure. 1 18

In conclusion, Gorlin‒Goltz syndrome is a rare hereditary disorder with autosomal dominant inheritance caused by variants in the hedgehog signaling pathway. While the diagnosis was made based on clinical findings in general, confirmation of the genetic variants makes an ideal diagnosis and suggests a new treatment method for targeted therapy.
